# First observation of ^28^O

**DOI:** 10.1038/s41586-023-06352-6

**Published:** 2023-08-30

**Authors:** Y. Kondo, N. L. Achouri, H. Al Falou, L. Atar, T. Aumann, H. Baba, K. Boretzky, C. Caesar, D. Calvet, H. Chae, N. Chiga, A. Corsi, F. Delaunay, A. Delbart, Q. Deshayes, Zs. Dombrádi, C. A. Douma, A. Ekström, Z. Elekes, C. Forssén, I. Gašparić, J.-M. Gheller, J. Gibelin, A. Gillibert, G. Hagen, M. N. Harakeh, A. Hirayama, C. R. Hoffman, M. Holl, A. Horvat, Á. Horváth, J. W. Hwang, T. Isobe, W. G. Jiang, J. Kahlbow, N. Kalantar-Nayestanaki, S. Kawase, S. Kim, K. Kisamori, T. Kobayashi, D. Körper, S. Koyama, I. Kuti, V. Lapoux, S. Lindberg, F. M. Marqués, S. Masuoka, J. Mayer, K. Miki, T. Murakami, M. Najafi, T. Nakamura, K. Nakano, N. Nakatsuka, T. Nilsson, A. Obertelli, K. Ogata, F. de Oliveira Santos, N. A. Orr, H. Otsu, T. Otsuka, T. Ozaki, V. Panin, T. Papenbrock, S. Paschalis, A. Revel, D. Rossi, A. T. Saito, T. Y. Saito, M. Sasano, H. Sato, Y. Satou, H. Scheit, F. Schindler, P. Schrock, M. Shikata, N. Shimizu, Y. Shimizu, H. Simon, D. Sohler, O. Sorlin, L. Stuhl, Z. H. Sun, S. Takeuchi, M. Tanaka, M. Thoennessen, H. Törnqvist, Y. Togano, T. Tomai, J. Tscheuschner, J. Tsubota, N. Tsunoda, T. Uesaka, Y. Utsuno, I. Vernon, H. Wang, Z. Yang, M. Yasuda, K. Yoneda, S. Yoshida

**Affiliations:** 1 Department of Physics, Tokyo Institute of Technology, Tokyo, Japan; 2 RIKEN Nishina Center, Saitama, Japan; 3 LPC Caen UMR6534, Université de Caen Normandie, ENSICAEN, CNRS/IN2P3, Caen, France; 4 Lebanese University, Beirut, Lebanon; 5 Lebanese-French University of Technology and Applied Sciences, Deddeh, Lebanon; 6 Institut für Kernphysik, Technische Universität Darmstadt, Darmstadt, Germany; 7 GSI Helmholtzzentrum für Schwerionenforschung, Darmstadt, Germany; 8 Helmholtz Research Academy Hesse for FAIR, Darmstadt, Germany; 9 Irfu, CEA, Université Paris-Saclay, Gif-sur-Yvette, France; 10 Institute for Basic Science, Daejeon, Republic of Korea; 11 Atomki, Debrecen, Hungary; 12 ESRIG, University of Groningen, Groningen, The Netherlands; 13 Institutionen för Fysik, Chalmers Tekniska Högskola, Göteborg, Sweden; 14 Ruđer Bošković Institute, Zagreb, Croatia; 15 Physics Division, Oak Ridge National Laboratory, Oak Ridge, TN USA; 16 Department of Physics and Astronomy, University of Tennessee, Knoxville, TN USA; 17 Physics Division, Argonne National Laboratory, Argonne, IL USA; 18 Eötvös Loránd University, Budapest, Hungary; 19 Center for Exotic Nuclear Studies, Institute for Basic Science, Daejeon, Republic of Korea; 20 Department of Physics and Astronomy, Seoul National University, Seoul, Republic of Korea; 21 Department of Advanced Energy Engineering Science, Kyushu University, Fukuoka, Japan; 22 Department of Physics, Tohoku University, Miyagi, Japan; 23 Department of Physics, The University of Tokyo, Tokyo, Japan; 24 Center for Nuclear Study, The University of Tokyo, Saitama, Japan; 25 Institut für Kernphysik, Universität zu Köln, Köln, Germany; 26 Department of Physics, Kyoto University, Kyoto, Japan; 27 Department of Physics, Kyushu University, Fukuoka, Japan; 28 Research Center for Nuclear Physics, Osaka University, Osaka, Japan; 29 Department of Physics, Osaka City University, Osaka, Japan; 30 Grand Accélérateur National d’Ions Lourds (GANIL), CEA/DRF-CNRS/IN2P3, Caen, France; 31 Center for Computational Sciences, University of Tsukuba, Ibaraki, Japan; 32 Department of Physics, Osaka University, Osaka, Japan; 33 Facility for Rare Isotope Beams, Michigan State University, East Lansing, MI USA; 34 Department of Physics, Rikkyo University, Tokyo, Japan; 35 Advanced Science Research Center, Japan Atomic Energy Agency, Ibaraki, Japan; 36 Department of Mathematical Sciences, Durham University, Durham, UK; 37 Liberal and General Education Center, Institute for Promotion of Higher Academic Education, Utsunomiya University, Tochigi, Japan

**Keywords:** Experimental nuclear physics, Nuclear physics

## Abstract

Subjecting a physical system to extreme conditions is one of the means often used to obtain a better understanding and deeper insight into its organization and structure. In the case of the atomic nucleus, one such approach is to investigate isotopes that have very different neutron-to-proton (*N*/*Z*) ratios than in stable nuclei. Light, neutron-rich isotopes exhibit the most asymmetric *N*/*Z* ratios and those lying beyond the limits of binding, which undergo spontaneous neutron emission and exist only as very short-lived resonances (about 10^−21^ s), provide the most stringent tests of modern nuclear-structure theories. Here we report on the first observation of ^28^O and ^27^O through their decay into ^24^O and four and three neutrons, respectively. The ^28^O nucleus is of particular interest as, with the *Z* = 8 and *N* = 20 magic numbers^1,2^, it is expected in the standard shell-model picture of nuclear structure to be one of a relatively small number of so-called ‘doubly magic’ nuclei. Both ^27^O and ^28^O were found to exist as narrow, low-lying resonances and their decay energies are compared here to the results of sophisticated theoretical modelling, including a large-scale shell-model calculation and a newly developed statistical approach. In both cases, the underlying nuclear interactions were derived from effective field theories of quantum chromodynamics. Finally, it is shown that the cross-section for the production of ^28^O from a ^29^F beam is consistent with it not exhibiting a closed *N* = 20 shell structure.

## Abstract (web summary)

Observation of ^28^O and ^27^O through their decay into ^24^O and four and three neutrons, respectively, is reported, with the ^28^O nucleus being of particular interest owing to proton and neutron magic numbers and its extremely asymmetric neutron-to-proton ratio.

## Main

One of the most active areas of present-day nuclear physics is the investigation of rare isotopes with large *N*/*Z* imbalances. The structure of such nuclei provides for strong tests of our theories, including—most recently—sophisticated ab initio-type approaches whereby the underlying interactions between the constituent nucleons are constructed from first-principles approaches (see, for example, ref. ^3^).

**Fig. 1:**
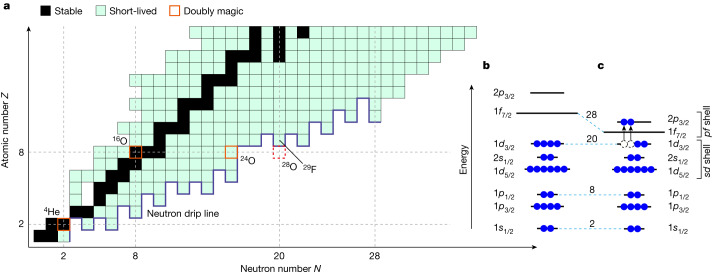
Nuclear chart and shell structure. **a**, Nuclear chart up to *Z* = 18 showing the stable and short-lived (β-decaying) nuclei. The experimentally established neutron drip line is shown by the thick blue line. Known doubly magic nuclei are also indicated. **b**, Schematic illustration of the neutron configuration for a nucleus with a closed *N* = 20 shell. **c**, The neutron configuration involving particle–hole excitations across a quenched *N* = 20 shell gap.

Owing to the strong nuclear force, nuclei remain bound to the addition of many more neutrons than protons and the most extreme *N*/*Z* asymmetries are found for light, neutron-rich nuclei (Fig. 1a). Here, beyond the limits of nuclear binding—the so-called neutron drip line—nuclei can exist as very-short-lived (about 10^−21^ s) resonances, which decay by spontaneous neutron emission, with their energies and lifetimes dependent on the underlying structure of the system. Experimentally, such nuclei can only be reached for the lightest systems (Fig. 1a), in which the location of the neutron drip line has been established up to neon (*Z* = 10)^4^ and the heaviest neutron unbound nucleus observed for fluorine (*Z* = 9) ^28^F (ref. ^5^). Arguably the most extreme system, if confirmed to exist as a resonance, would be the tetra-neutron, for which a narrow near-threshold continuum structure has been found in a recent missing-mass measurement^6^. Here we report on the direct observation of ^28^O (*N*/*Z* = 2.5), which is unbound to four-neutron decay, and of neighbouring ^27^O (three-neutron unbound).

The nucleus ^28^O has long been of interest^7,8^ as, in the standard shell-model picture of nuclear structure, it is expected to be ‘doubly magic’. Indeed, it is very well established that for stable and near-stable nuclei, the proton and neutron numbers 2, 8, 20, 28, 50, 82 and 126 correspond to spherical closed shells^1,2^. Such nuclei represent a cornerstone in our understanding of the structure of the many-body nuclear system. In particular, as substantial energy is required to excite them owing to the large shell gaps, they can be considered, when modelling nuclei in their mass region, as an ‘inert’ core with no internal degrees of freedom. Such an approach has historically enabled more tractable calculations to be made than attempting to model an *A*-body (*A* = *Z* + *N*) nucleus from the full ensemble of nucleons. Indeed, this approach has been a fundamental premise of the shell-model methods that have enabled an extremely wide variety of structural properties of nuclei to be described with good accuracy over several decades (see, for example, ref. ^9^).

Of the very limited number of nuclei that are expected to be doubly magic based on the classical shell closures, ^28^O is, given its extreme *N*/*Z* asymmetry, the only one that is—in principle—experimentally accessible that has yet to be observed. In recent years, the doubly magic character of the two other such neutron-rich nuclei, ^78^Ni (*Z* = 28, *N* = 50; *N*/*Z* = 1.8)^10^ and ^132^Sn (*Z* = 50, *N* = 82; *N*/*Z* = 1.6)^11^, has been confirmed. The remaining candidate, two-neutron unbound nucleus ^10^He (*Z* = 2, *N* = 8; *N*/*Z* = 4), has been observed as a well-defined resonance but its magicity or otherwise has yet to be established (ref. ^12^, and references therein).

The *N* = 20 shell closure has long been known, however, to disappear in the neutron-rich Ne, Na and Mg (*Z* = 10–12) isotopes (see, for example, refs. ^13,14^). This region is referred to as the ‘Island of Inversion’ (IoI)^15^, whereby the energy gap between the neutron *sd*-shell and *pf*-shell orbitals, rather than being well pronounced (Fig. 1b), is weakened or even vanishes, and configurations with neutrons excited into the *pf*-shell orbitals dominate the ground state (gs) of these nuclei, as shown schematically in Fig. 1c. The IoI nuclei with such configurations are well deformed, rather than spherical, and exhibit low-lying excited states. Very recently, the IoI has been shown to extend to the fluorine isotopes ^28,29^F (*N* = 19, 20)^5,16–18^ that neighbour ^28^O. On the other hand, the last particle-bound oxygen isotope, ^24^O, has been found to be doubly magic, with a new closed shell forming at *N* = 16 (refs. ^19–23^). As such, the structural character of the more neutron-rich oxygen isotopes and, in particular, ^28^O is an intriguing question. So far, however, only ^25,26^O (*N* = 17, 18) have been observed, as single-neutron and two-neutron unbound systems, respectively^24–27^, with the latter existing as an extremely narrow, barely unbound resonance.

This investigation focused on the search for ^27,28^O, produced in high-energy reactions, through the direct detection of their decay products—^24^O and three or four neutrons. Critical to the success of this work was the capability of the RIKEN RI Beam Factory to produce intense neutron-drip-line beams coupled to a thick, active liquid-hydrogen target system and a high-performance multineutron detection array.

## Experiment

**Extended Data Fig. 1:**
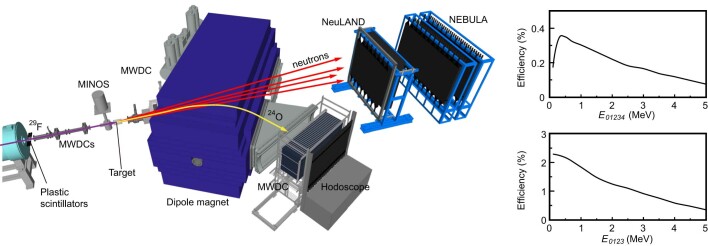
Schematic view of the experimental setup. The insets show the overall efficiency as a function of decay energy for detecting ^24^O and four and three neutrons.

The neutron-unbound ^27,28^O were produced through proton-induced nucleon knockout reactions from a 235 MeV per nucleon beam of ^29^F. As depicted in Extended Data Fig. 1, the ^29^F ions were characterized and tracked onto a thick (151 mm) liquid-hydrogen reaction target using plastic scintillators and multiwire drift chambers. The hydrogen target was surrounded by the MINOS Time Projection Chamber^28^, which allowed for the determination of the reaction vertex. This combination provided for both the maximum possible luminosity together with the ability to maintain a good ^27,28^O decay-energy resolution.

The forward-focused beam-velocity reaction products—charged fragments and fast neutrons—were detected and their momenta determined using the SAMURAI spectrometer^29^, including the three large-area segmented plastic scintillator walls of the NeuLAND^30^ and NEBULA arrays. An overall detection efficiency for the three-neutron and four-neutron decay of around 2% and 0.4%, respectively, was achieved for decay energies of 0.5 MeV (Extended Data Fig. 1). The decay energies were reconstructed from the measured momenta using the invariant-mass technique with a resolution (full width at half maximum, FWHM) of around 0.2 MeV at 0.5 MeV decay energy (see Methods).

## Analysis and results

The ^24^O fragments were identified by the magnetic rigidity, energy loss and time of flight derived from the SAMURAI spectrometer detectors. The neutrons incident on the NeuLAND and NEBULA arrays were identified on the basis of the time of flight and energy deposited in the plastic scintillators. Notably, the multineutron detection required the application of dedicated offline analysis procedures to reject crosstalk (see Methods), that is, events in which a neutron is scattered between and registered in two or more scintillators.

**Fig. 2:**
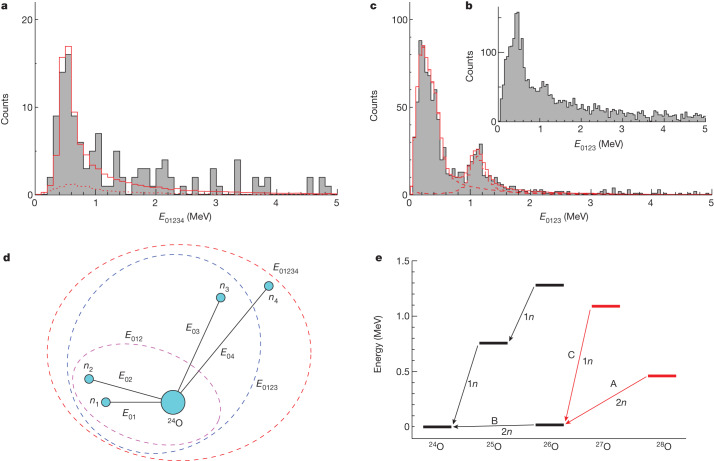
Decay-energy spectra and decay scheme. **a**, Five-body decay-energy (*E*_01234_) spectrum for ^24^O+4*n* events. The solid red histogram shows the best-fit result taking into account the experimental response function. The dotted red histogram shows the contribution arising from residual crosstalk that survives the rejection procedures (see Methods). **b**, Four-body decay-energy (*E*_0123_) spectrum for ^24^O+3*n* events. **c**, Same as **b** but gated by the partial decay energy *E*_012_ < 0.08 MeV. The dashed red histograms represent the contributions from ^28^O and ^27^O events and the solid red histogram shows the sum. **d**, Definition of the partial decay energies. **e**, Decay scheme of the unbound oxygen isotopes. The newly observed resonances and their decays are shown in red. Source Data

In the analysis, the decay neutrons were denoted *n*_1_, *n*_2_,… by ascending order of the two-body relative energy *E*_0*i*_ between ^24^O and *n*_*i*_, such that *E*_01_ < *E*_02_ < *E*_03_ < *E*_04_ (Fig. 2d). The ^28^O decay energy, *E*_01234_, reconstructed from the measured momentum vectors of the five decay particles, is shown in Fig. 2a. A narrow peak is clearly observed at about 0.5 MeV and may be assigned to be the ^28^O ground state. As a small fraction of crosstalk events could not be eliminated by the rejection procedures, care must be taken to understand their contribution to the *E*_01234_ spectrum. In particular, ^24^O+3*n* events, in which one of the neutrons creates crosstalk and is not identified as such in the analysis, can mimic true ^28^O decay. In this context, to provide a complete and consistent description of all the ^24^O+*xn* decay-energy spectra, a full Monte Carlo simulation was constructed (see Methods). As shown in Fig. 2a, the contribution from the residual crosstalk events is found to be rather limited in magnitude in the ^24^O+4*n* decay-energy spectrum and, moreover, produces a very broad distribution.

**Extended Data Fig. 2:**
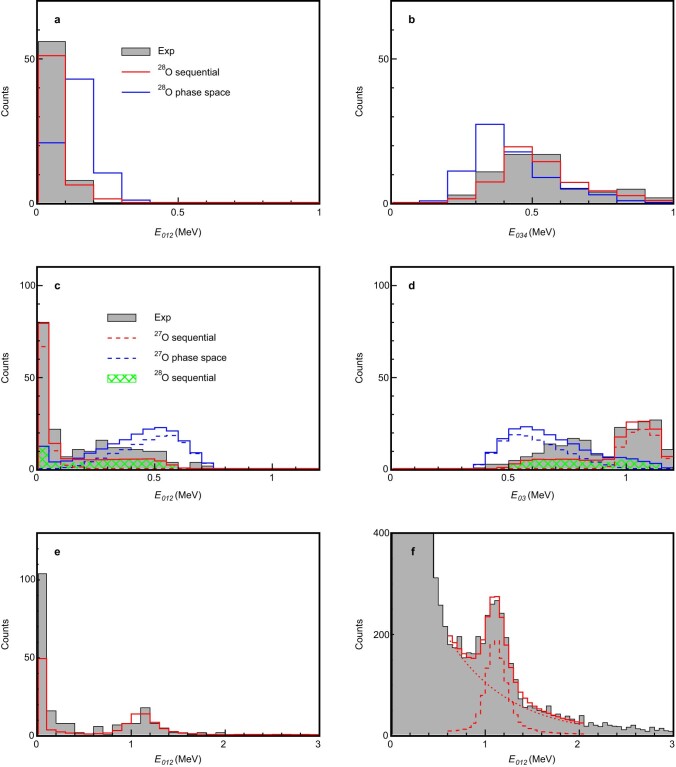
Partial decay-energy spectra. **a**, The filled grey histogram is the three-body decay energy *E*_012_ gated on the total decay energy *E*_01234_ < 1 MeV for the ^24^O+4*n* coincidence events. The red and blue histograms are the results of simulations of sequential decay through the ^26^O ground state (A and B in Fig. 2e) and five-body phase-space decay, respectively. **b**, Same as **a** but for the three-body decay energy *E*_034_. **c**, The filled grey histogram is the partial decay-energy spectrum *E*_012_ gated by 1.0 < *E*_0123_ < 1.2 MeV for the ^24^O+3*n* coincidence events. The red and blue dashed histograms are the results of simulations assuming ^27^O sequential (B and C in Fig. 2e) and four-body phase-space decay, respectively. The green hatched histogram represents the contribution from the decay of ^28^O. The red (blue) solid histogram is the sum of the contributions from ^28^O and ^27^O for sequential (phase-space) decay. **d**, Same as **c** but for the two-body decay energy *E*_03_. **e**, Decay-energy spectrum of ^24^O+2*n* events from the ^29^Ne beam data. The grey histogram represents events with *E*_01_ < 0.08 MeV. The red histogram shows the results of the simulation for the decay of the ^27^O resonance. The excess observed near-zero decay energy is interpreted as arising from direct population of the ^26^O ground state from ^29^Ne. **f**, Decay-energy spectrum of ^24^O+2*n* events from the ^29^F beam. The grey histogram represents events with *E*_01_ < 0.08 MeV. The red histogram shows the best fit in the region of the peak arising from the decay of the ^27^O resonance (dashed histogram) and an exponential distribution (dotted curve) arising from all other contributions that come primarily from the decay of ^28^O. Source Data

The decay of ^28^O was investigated by examining the correlations in the ^24^O plus neutrons subsystems (see Methods). In particular, the three-body (^24^O+*n*_1_+*n*_2_) partial decay energy *E*_012_ (Extended Data Fig. 2a) was reconstructed from the ^24^O+4*n* dataset. The corresponding spectrum exhibits a sharp threshold peak arising from ^26^O_gs_, which is known to have a decay energy of only 18(5) keV (ref. ^27^). This observation clearly indicates that ^28^O sequentially decays through ^26^O_gs_ as shown by the arrows A and B in Fig. 2e.

We have also observed, in the ^24^O+3*n* channel, a ^27^O resonance for the first time, as may be seen in the four-body decay-energy (*E*_0123_) spectrum of Fig. 2b. As confirmed by the simulations, which are able to simultaneously describe the ^24^O+3*n* and 4*n* decay-energy spectra, the well-populated peak-like structure below about 0.5 MeV corresponds to ^28^O events in which only three of the four emitted neutrons are detected. The peak at *E*_0123_ ≈ 1 MeV, however, cannot be generated by such events and must arise from a ^27^O resonance. This was confirmed by the analysis of the data acquired with a ^29^Ne beam (see Methods and Extended Data Fig. 2e), in which ^27^O can be produced by two-proton removal but not ^28^O, as this requires the addition of a neutron. The ^27^O resonance also decays sequentially through ^26^O_gs_, as shown by the arrows B and C in Fig. 2e from the analysis of the partial decay energies (Extended Data Fig. 2c,d).

The decay energies of the ^27,28^O resonances were derived from a fit of the *E*_0123_ spectrum with the condition that the partial decay energy satisfies *E*_012_ < 0.08 MeV (Fig. 2c), that is, decay through the ^26^O ground state was selected so as to minimize the uncertainties owing to contributions from higher-lying ^28^O resonances that were not identified in the present measurements owing to the limited detection efficiency (Extended Data Fig. 1). The fitting used line shapes that incorporated the effects of the experimental response functions, as derived from the simulations, including the contribution arising from the residual crosstalk (see Methods).

In the case of ^28^O, a decay energy of $${E}_{01234}={0.46}_{-0.04}^{+0.05}({\rm{stat}})\,\pm $$$$0.02({\rm{syst}})\,{\rm{MeV}}$$ was found, with an upper limit of the width of the resonance of 0.7 MeV (68% confidence interval). The cross-section for single-proton removal from ^29^F populating the resonance was deduced to be $${1.36}_{-0.14}^{+0.16}({\rm{stat}})\pm 0.13({\rm{syst}})\,{\rm{mb}}$$. The systematic uncertainties for the decay energy and the width were dominated by the precise conditions used in the neutron-crosstalk-rejection procedures, whereas the principal contribution to that for the cross-section arose from the uncertainty in the neutron-detection efficiency. It may be noted that, if the resonance observed here is an excited state of ^28^O (presumably the 2^+^ level), then the ground state must lie even closer to threshold and the excitation energy of the former must be less than 0.46 MeV. This, however, is very much lower than theory suggests (2 MeV or more), even when the *N* = 20 shell closure is absent (see below). As such, it is concluded that the ground state has been observed.

In the case of ^27^O, a decay energy of *E*_0123_ = 1.09 ± 0.04(stat) ± 0.02(syst) MeV was found. The width of the resonance was comparable with the estimated experimental resolution of 0.22 MeV (FWHM). Nevertheless, it was possible to obtain an upper limit on the width—0.18 MeV (68% confidence interval)—through a fit of a gated *E*_012_ spectrum for the much higher statistics ^24^O and two-neutron coincidence events, as shown in Extended Data Fig. 2f. The spin and parity (*J*^π^) of the resonance may be tentatively assigned to be 3/2^+^ or 7/2^−^ based on the upper limit of the width (see Methods).

## Comparison with theory

**Fig. 3:**
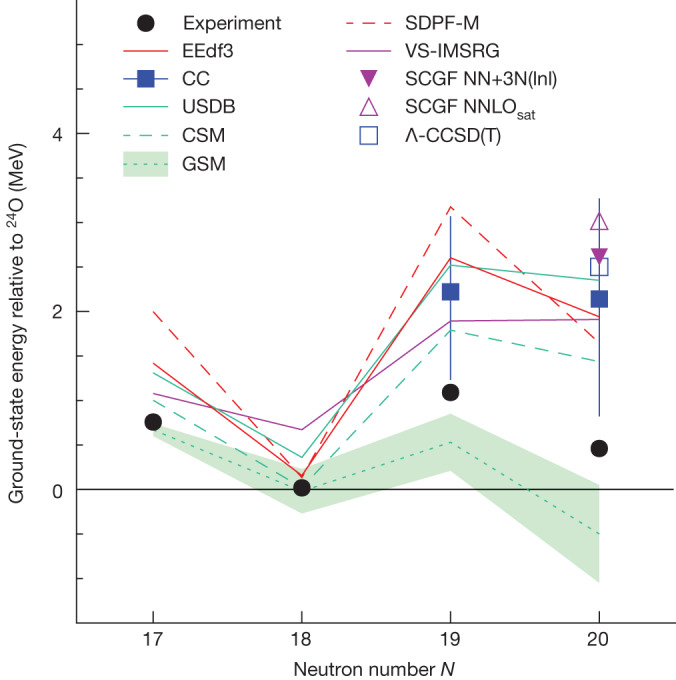
Ground-state energies with respect to ^24^O. Experiment is shown by the black circles, in which the values for ^27,28^O are the present results and those for ^25,26^O are taken from the atomic mass evaluation^54^. The experimental uncertainties are smaller than the symbol size. Comparison is made with predictions of shell-model calculations using the EEdf3 (refs. ^31,32^), USDB^9^ and SDPF-M^37^ (see text for ^27^O) interactions, the coupled-cluster method with the statistical approach (CC) and shell-model calculations incorporating continuum effects (CSM^38^ and GSM^39^). Also shown are the predictions of ab initio approaches (VS-IMSRG^33^, SCGF^35^ and Λ-CCSD(T)^36^). The vertical bars for CC denote 68% credible intervals. The shaded band for GSM shows the uncertainties owing to *pf*-continuum couplings.

The experimental ground-state energies of the oxygen isotopes ^25–28^O are summarized in Fig. 3 and compared with theoretical calculations based on chiral effective field theory (*χ*EFT)^31–36^ and large-scale shell-model calculations^9,37^, including those with continuum effects^38,39^. We focus on large-scale shell-model and coupled-cluster calculations, in which the latter is augmented with a new statistical method. Both techniques include explicitly three-nucleon forces, which are known to play a key role in describing the structure of neutron-rich nuclei, including the oxygen isotopes and the location of the *Z* = 8 neutron drip line at ^24^O (refs. ^40–42^).

The large-scale shell-model calculations were undertaken using the new EEdf3 interaction, which was constructed on the basis of *χ*EFT (see Methods). Because the calculations use a model space that includes the *pf*-shell orbitals, the disappearance of the *N* = 20 shell closure can be naturally described. The EEdf3 interaction is a modified version of EEdf1 (refs. ^31,32^), which correctly predicts the neutron drip line at F, Ne and Na, as well as a relatively low-lying ^29^F excited state^17^ and the appreciable occupancy of the neutron 2*p*_3/2_ orbital^5,18^. The EEdf3 interaction, which includes the effects of the EFT three-nucleon forces^43^, provides a reasonable description of the trends in the masses of the oxygen isotopes. However, as may be seen in Fig. 3, it predicts slightly higher ^27,28^O energies (about 1 MeV) than found in the experiment. The calculated sum of the occupation numbers for the neutron *pf*-shell orbitals is 2.5 (1.4) for ^28^O (^27^O) and for the 1*d*_3/2_ orbital 2.0 (2.1), which are consistent with a collapse of the *N* = 20 shell closure. The EEdf3 calculations show that ^28^O_gs_ has large admixtures of configurations involving neutron excitations in the *pf*-shell orbitals, as expected for nuclei in the IoI. This is supported by the measured cross-section as discussed below.

**Extended Data Fig. 3:**
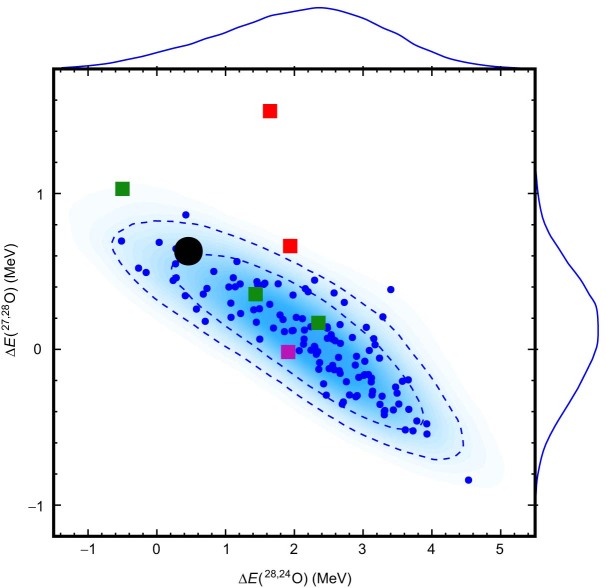
Probability distribution of the calculated energy differences. Survived non-implausible calculations are shown by blue dots as functions of energy differences Δ*E*(^28,24^O) and Δ*E*(^27,28^O). The black circle shows experiment. The dashed curves indicate 68% and 90% highest probability density regions. The top and right distributions are the one-dimensional probability density distributions. The values given by the other theories are plotted as squares: green, USDB, GSM and CSM; red, SDPF-M and EEdf3; purple, VS-IMSRG.

First-principles calculations were performed using the coupled-cluster (CC) method guided by history matching (HM)^44–46^ to explore the parameter space of the 17 low-energy constants (LECs) in the *χ*EFT description of the two-nucleon and three-nucleon interactions. HM identifies the region of parameter space for which the emulated CC method generates non-implausible results (see Methods). A reliable, low-statistic sample of 121 different LEC parameterizations was extracted, for which the CC posterior predictive distribution (ppd) was computed for the ground-state energies of ^27,28^O, which are shown in Fig. 3. The predicted ^27,28^O energies are correlated, as is clearly seen in the plot of energy distributions shown in Extended Data Fig. 3. From this, the median values and 68% credible regions were obtained for the ^27^O–^28^O and ^28^O–^24^O energy differences: $\Delta E({}^{27,28}{\rm{O}})={0.11}_{+0.36}^{-0.39}\,{\rm{M}}{\rm{e}}{\rm{V}}$ and $\Delta E{(}^{28,24}{\rm{O}})={2.1}_{+1.2}^{-1.3}\,{\rm{M}}{\rm{e}}{\rm{V}}$. The experimental values Δ*E*(^27,28^O) = 0.63 ± 0.06(stat) ± 0.03(syst) MeV and $\Delta E{(}^{28,24}{\rm{O}})={0.46}_{-0.04}^{+0.05}({\rm{s}}{\rm{t}}{\rm{a}}{\rm{t}})\pm 0.02({\rm{s}}{\rm{y}}{\rm{s}}{\rm{t}})\,{\rm{M}}{\rm{e}}{\rm{V}}$, located at the edge of the 68% credible region, are consistent with the CC ppd. However, it is far enough away from the maximum to suggest that only a few finely tuned chiral interactions may be able to reproduce the ^27^O and ^28^O energies. Also, the obtained credible regions of the ^27,28^O energies with respect to ^24^O are relatively large, demonstrating that the measured decay energies of the extremely neutron-rich isotopes ^27,28^O are valuable anchors for theoretical approaches based on *χ*EFT.

In Fig. 3, the predictions of a range of other models are shown. The USDB^9^ effective interaction (constructed within the *sd* shell) provides for arguably the most reliable predictions of the properties of *sd*-shell nuclei. The continuum shell model (CSM)^38^ and the Gamow shell model (GSM)^39^ include the effects of the continuum, which should be important for drip-line and unbound nuclei. The shell-model calculation using the SDPF-M interaction^37^ includes the *pf*-shell orbitals in its model space, which should be important if either or both ^27,28^O lie within the IoI. All the calculations, except those with the SDPF-M interaction, predict a *J*^π^ = 3/2^+^ ^27^O_gs_. In the case of the SDPF-M, a 3/2^−^ ground state is found with essentially degenerate 3/2^+^ (energy plotted in Fig. 3) and 7/2^−^ excited states at 0.71 MeV.

The remaining theoretical predictions are based on *χ*EFT interactions. The valence-space in-medium similarity renormalization group (VS-IMSRG)^33^ uses the 1.8/2.0 (EM) EFT potential^43^. The results for the self-consistent Green’s function (SCGF) approach are shown for the NNLO_sat_ (ref. ^47^) and NN+3N(lnl) potentials^35^. The coupled-cluster calculation (Λ-CCSD(T)^36^) using NNLO_sat_ is also shown. Except for the results obtained using the GSM, all of the calculations shown predict higher energies than found here for ^27^O and ^28^O.

**Extended Data Fig. 4:**
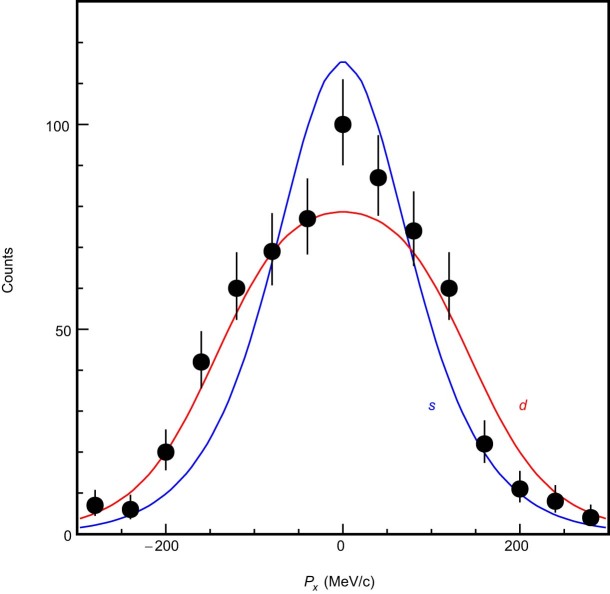
Transverse momentum distribution of the ^24^O+3*n* system in the rest frame of the ^29^F beam. Events corresponding to the population of the ^28^O ground state (*E*_012_ < 0.08 MeV and *E*_0123_ < 0.8 MeV) are shown by the data points. The blue and red solid lines represent the DWIA calculations, including the experimental effects for *s*_1/2_ and *d*_5/2_ proton knockout, respectively, whereby the distributions have been scaled to best fit experiment. Source Data

We now turn to the question of whether the *N* = 20 shell closure occurs in ^28^O. Specifically, the measured cross-section for single-proton removal from ^29^F may be used to deduce the corresponding spectroscopic factor (*C*^2^*S*), which is a measure of the degree of overlap between initial and final state wavefunctions. As noted at the start of this paper, the *N* = 20 shell closure disappears in ^29^F and the ground state is dominated by neutron *pf*-shell configurations^5,16–18^. As such, if the neutron configuration of ^28^O is very similar to ^29^F and the *Z* = 8 shell closure is rigid, the spectroscopic factor for proton removal will be close to unity. The spectroscopic factor was deduced using the distorted-wave impulse approximation (DWIA) approach (see Methods). As recent theoretical calculations predict *J*^π^ = 5/2^+^ or 1/2^+^ for ^29^F_gs_ (see, for example, refs. ^5,31,32,48–50^), the momentum distribution has been investigated (Extended Data Fig. 4) and was found to be consistent with proton removal from the 1*d*_5/2_ orbital (see Methods), leading to a 5/2^+^ assignment. The ratio of the measured to theoretical single-particle cross-section provides for an experimentally deduced spectroscopic factor of ${C}^{2}S={0.48}_{-0.06}^{+0.05}({\rm{stat}})\pm 0.05({\rm{syst}})$. Such an appreciable strength indicates that the ^28^O neutron configuration resembles that of ^29^F. This value may be compared with that of 0.68 derived from the EEdf3 shell-model calculations (in which the centre-of-mass correction factor^51^ (29/28)^2^ has been applied). The 30% difference between the experimental *C*^2^*S* as compared with theory is in line with the well-known reduction factor observed in (*p*, 2*p*) and (*e*, *e*′*p*) reactions^52^. Notably, the EEdf3 calculations predict admixtures of the ground-state wavefunction of ^29^F with *sd*-closed-shell configurations of only 12%. Consequently, even when the neutrons in ^28^O are confined to the *sd* shell, a spectroscopic factor of only 0.13 is obtained. As such, it is concluded that, as in ^29^F, the *pf*-shell neutron configurations play a major role in ^28^O and that the *N* = 20 shell closure disappears. Consequently, the IoI extends to ^28^O and it is not a doubly magic nucleus.

More effort will be required to properly quantify the character of the structure of ^28^O and the neutron *pf*-shell configurations. In this context, the determination of  the excitation energy of the first 2^+^ state is the next step that may be deduced experimentally^17^. The EEdf3 calculations predict an excitation energy of 2.097 MeV, which is close to that of approximately 2.5 MeV computed by the particle rotor model assuming moderate deformation^53^. Both predictions are much lower than the energies found in doubly magic nuclei, for example, 6.917 MeV in ^16^O and 4.7 MeV in ^24^O (refs. ^21,23^). A complementary probe of the neutron *sd*–*pf* shell gap, which is within experimental reach, is the energy difference between the positive-parity and negative-parity states of ^27^O as seen in ^28^F (ref. ^5^).

## Conclusions

We have reported here on the first observation of the extremely neutron-rich oxygen isotopes ^27,28^O. Both nuclei were found to exist as relatively low-lying resonances. These observations were made possible using a state-of-the-art setup that permitted the direct detection of three and four neutrons. From an experimental point of view, the multineutron-decay spectroscopy demonstrated here opens up new perspectives in the investigation of other extremely neutron-rich systems lying beyond the neutron drip line and the study of multineutron correlations. Comparison of the measured energies of ^27,28^O with respect to ^24^O with a broad range of theoretical predictions, including two approaches using nuclear interactions derived from effective field theories of quantum chromodynamics, showed that—in almost all cases—theory underbinds both systems. The statistical coupled-cluster calculations indicated that the energies of ^27,28^O can provide valuable constraints of such ab initio approaches and, in particular, the interactions used. Finally, although ^28^O is expected in the standard shell-model picture to be a doubly magic nucleus (*Z* = 8 and *N* = 20), the single-proton removal cross-section measured here, when compared with theory, was found to be consistent with it not having a closed neutron shell character. This result suggests that the IoI extends beyond ^28,29^F into the oxygen isotopes.

## Methods

### Production of the ^29^F beam

The beam of ^29^F ions was provided by the RI Beam Factory operated by the RIKEN Nishina Center and the Center for Nuclear Study, University of Tokyo. It was produced by projectile fragmentation of an intense 345-MeV-per-nucleon ^48^Ca beam on a 15-mm-thick beryllium target. The secondary beam, including ^29^F, was prepared using the BigRIPS^55^ fragment separator operated with aluminium degraders of 15 mm and 7 mm median thicknesses at the first and fifth intermediate focal planes, respectively. The primary ^48^Ca beam intensity was typically 3 × 10^12^ particles per second. The average intensity of the ^29^F beam was 90 particles per second.

### Measurement with a ^29^Ne beam

Data were also acquired to measure the direct population of ^27^O through two-proton removal from ^29^Ne. The beam was produced in a similar manner to that for ^29^F and the energy was 228 MeV per nucleon with an average intensity of 8 × 10^3^ particles per second.

Unfortunately, in this measurement, the cross-section for the two-proton removal was much lower than expected and the statistics obtained for ^24^O+3*n* coincidence events was too low to be usefully exploited. Nevertheless, the decay of ^27^O could be identified from the ^24^O+2*n* coincidence data. As may be seen in Extended Data Fig. 2e, the three-body decay-energy (*E*_012_) spectrum gated by *E*_01_ < 0.08 MeV, corresponding to selection of the ^26^O ground-state decay, exhibits a clear peak at around 1 MeV. As the simulations demonstrate, this is consistent with the sequential decay of the ^27^O resonance observed in the ^29^F beam data (Fig. 2c).

### Invariant-mass method

The invariant mass of ^28^O, *M*(^28^O), was reconstructed from the momentum vectors of all the decay particles (^24^O and 4*n*) with $M({}^{28}{\rm{O}})=\sqrt{{(\sum {E}_{i})}^{2}-{|\sum {{\bf{p}}}_{i}|}^{2}}$, in which *E*_*i*_ and **p**_*i*_ denote the total energy and momentum vector of the decay particles, respectively. The decay energy is then obtained as *E*_01234_ = *M*(^28^O) − *M*(^24^O) − 4*M*_*n*_, in which *M*(^24^O) and *M*_*n*_ are the masses of ^24^O and the neutron, respectively. The decay-energy resolution is estimated by Monte Carlo simulations. The resolution (FWHM) varies as a function of the decay energy approximately as 0.14(*E*_01234_ + 0.87)^0.81^ MeV.

### Simulations

The experimental response functions, for both the full and partial decay-energy spectra, were derived from a Monte Carlo simulation based on GEANT4 (ref. ^56^). All relevant characteristics of the setup (geometrical acceptances and detector resolutions) were incorporated, as well as those of the beam, target and reaction effects. The QGSP_INCLXX physics class was used to describe the interactions of the neutrons in the detectors (as well as non-active material), as it reproduces well the experimentally determined single-neutron detection efficiency as well as the detailed characteristics of neutron crosstalk events^57,58^. The generated events were treated using the same analysis procedure as for the experimental data. The overall efficiency as a function of decay energy for detecting ^24^O and three and four neutrons, as estimated by the simulations, is shown by the insets of Extended Data Fig. 1.

### Fitting of decay-energy spectra

The energies, widths and amplitudes of the resonances, as modelled by intrinsic line shapes with a Breit–Wigner form with energy-dependent widths, were obtained through fits of the corresponding decay-energy spectra using the maximum-likelihood method, in which the experimental responses were obtained by the simulations. As the decays of both ^27^O and ^28^O proceed through the ^26^O ground state (18 keV (ref. ^27^)), the width of which is very small, the observed widths will be dominated by the one-neutron and two-neutron decay, respectively, to ^26^O. We assume an ${E}_{01234}^{2}$ dependence of the width for the 2*n* emission^59^ to ^26^O in the case of ^28^O and an energy dependence for the width of the single-neutron emission^60^ from ^27^O to ^26^O. Fits with orbital angular momentum (*L*) dependent widths (*L* = 2 and 3) for the latter gave consistent results within the statistical uncertainties.

A non-resonant component is not included in the fitting as it is small, if not negligible, as in the cases of ^25,26^O produced in one-proton-removal reactions in previous experiments^24–27^. The event selection with *E*_012_ < 0.08 MeV should further reduce any such contribution. As a quantitative check, a fit with a non-resonant component—modelled with a line shape given by ${p}_{0}\sqrt{{E}_{0123}}\exp \left(-{p}_{1}{E}_{0123}\right)$, in which *p*_0_ and *p*_1_ are fitting parameters—has been examined. This gives 8% reduction in the ^28^O cross-section with a very limited impact on the energies and widths of the ^27,28^O resonances.

### Neutron crosstalk

A single beam-velocity neutron may scatter between individual plastic scintillator detectors of the three neutron walls of the setup. Such crosstalk events can mimic true multineutron events and present a source of background. By examining the apparent kinematics of such events and applying so-called causality conditions, this background can be almost completely eliminated^57,58^. Notably, both the rejection techniques and the rate and characteristics of the crosstalk have been benchmarked in and compared with the simulations for dedicated measurements with single-neutron beams.

In the case of the four-neutron detection to identify ^28^O, only 16% of the events arise from crosstalk that could not be eliminated (Fig. 2a). Most of these residual crosstalk events arise in cases in which one (or occasionally more) of the neutrons emitted in the decay of ^28^O is subject to crosstalk. A much smaller fraction is also estimated to be produced when one of the three neutrons from the decay of ^27^O, produced directly by proton and neutron knockout, undergoes crosstalk. Notably, the crosstalk cannot generate a narrow peak-like structure in the *E*_01234_ decay-energy spectrum.

### Partial decay energy of subsystems

The partial decay energies of the ^24^O+*xn* subsystems can be used to investigate the manner in which ^27,28^O decay. In this analysis, the decay neutrons are numbered (*n*_1_, *n*_2_,…) by ascending order of two-body relative energy *E*_0*i*_ between ^24^O and *n*_*i*_, that is, such that, *E*_01_ < *E*_02_ < *E*_03_ < *E*_04_. Of particular interest here is the extremely low decay energy of the ^26^O ground state (18 keV (ref. ^27^)), such that it appears just above zero energy (or the neutron-decay threshold) in the two-body partial decay energy *E*_01_ and three-body partial decay energy (*E*_012_).

Extended Data Fig. 2a,b shows the distributions of the partial decay energies *E*_012_ and *E*_034_ for the ^24^O+4*n* coincidence events with a total decay energy *E*_01234_ < 1 MeV. The resulting sharp threshold peak in the *E*_012_ spectrum is a clear sign of sequential decay through the ^26^O ground state. This is confirmed quantitatively by a simulation assuming two-neutron emission to the ^26^O ground state, which—in turn—decays by two-neutron emission to the ^24^O ground state, which describes well the *E*_012_ and *E*_034_ spectra. By comparison, a simulation assuming five-body phase-space decay fails to reproduce both of these spectra. We thus conclude that the ^28^O ground state sequentially decays through the ^26^O ground state as depicted in Fig. 2e.

In a similar vein, the sequential decay of ^27^O through the ^26^O ground state was identified from the analysis of the partial decay energies for the ^24^O+3*n* coincidence events. Extended Data Fig. 2c,d show the distributions of the partial decay energies *E*_012_ and *E*_03_ for events for which 1.0 < *E*_0123_ < 1.2 MeV. The *E*_012_ spectrum exhibits a strong enhancement at zero energy indicative of sequential decay through the ^26^O ground state. This interpretation is confirmed by the comparison shown with a simulation for the sequential decay of ^27^O including the contribution from the decay of ^28^O.

### Widths of the ^27,28^O resonances

As the energy of the ^28^O resonance is lower than those of ^27^O and ^25^O (Fig. 2e), both one-neutron and three-neutron emission are energetically forbidden. The two-neutron decay to the ^26^O ground state and the four-neutron decay to ^24^O are allowed with nearly equal decay energies. The former decay should be favoured as the effective few-body centrifugal barrier increases according to the number of emitted particles^59^. It may be noted that the upper limit of 0.7 MeV observed here for the ^28^O resonance width is consistent with the theoretical estimates for its sequential decay^59^.

The upper limit for the ^27^O width (0.18 MeV) may be compared with the single-particle widths^61^ for neutron decay. Because the width of ^26^O is very narrow owing to the extremely small decay energy (18 keV (ref. ^27^)), the ^27^O width should be dominated by that for the first step ^27^O → ^26^O+*n*. The widths for *s*-wave, *p*-wave, *d*-wave and *f*-wave neutron emission are 5, 3, 0.8 and 0.06 MeV, respectively. Assuming that the corresponding spectroscopic factors are not small (≳0.1), this would suggest that the decay occurs through *d*-wave or *f*-wave neutron emission. As such, the spin and parity of the ^27^O resonance may be tentatively assigned to be 3/2^+^ or 7/2^−^.

### Momentum distribution

Extended Data Fig. 4 shows the transverse momentum (*P*_*x*_) distribution of the ^24^O+3*n* system in the rest frame of the ^29^F beam for events gated by *E*_012_ < 0.08 MeV and *E*_0123_<0.8 MeV, that is, events corresponding to population of the ^28^O ground state. We note that this analysis used the ^24^O+3*n* events, as the limited ^24^O+4*n* statistics could not be usefully exploited in distinguishing between the momentum distributions for the proton knockout from different orbitals. Even though the momentum distribution is slightly broadened by the undetected decay neutron, it still reflects directly the character of the knocked out proton.

The experimental *P*_*x*_ distribution is compared with DWIA reaction theory calculations (see below) for knockout of a proton from the 1*d*_5/2_ and 2*s*_1/2_ orbitals. The theoretical distributions are convoluted with the experimental resolution, as well as the much smaller broadening induced by the undetected neutron (*σ* = 34 MeV/*c*). The best-fit normalization of the theoretical distribution obtained by the distorting potential with the Dirac phenomenology (microscopic folding-model potential) through a *χ*^2^ minimization gives reduced-*χ*^2^ values of 2.0 (2.0) for the 1*d*_5/2_ proton knockout and 3.7 (4.7) for the 2*s*_1/2_ knockout. The curves in Extended Data Fig. 4 represent the calculations obtained by the distorting potential with the Dirac phenomenology. The better agreement for the 1*d*_5/2_ proton knockout suggests that the spin and parity of the ^29^F ground state is 5/2^+^, as predicted by the shell-model calculations, including those using the EEdf3 interaction.

### EEdf3 calculations

The EEdf3 Hamiltonian^31^ is a variant of the EEdf1 Hamiltonian, which was used in ref. ^32^ for describing F, Ne, Na and Mg isotopes up to the neutron drip line^31^. The EEdf1 Hamiltonian was derived from *χ*EFT interaction, as described below. The *χ*EFT interaction proposed by Entem and Machleidt^62,63^ was taken with Λ = 500 MeV, as the nuclear force in vacuum, up to the next-to-next-to-next-to-leading-order (N^3^LO) in the *χ*EFT. It was then renormalized using the *V*_low-k_ approach^64,65^ with a cutoff of ${\Lambda }_{{V}_{{\rm{low}} \mbox{-} k}}=2.0\,{{\rm{fm}}}^{-1}$, to obtain a low-momentum interaction decoupled from high-momentum phenomena. The EKK method^66–68^ was then used to obtain the effective NN interaction for the *sd*–*pf* shells, by including the so-called $\hat{Q}$-box, which incorporates unfolded effects coming from outside the model space^69^, up to the third order and its folded diagrams. As to the single-particle basis vectors, the eigenfunctions of the three-dimensional harmonic oscillator potential were taken as usual. Also, the contributions from the Fujita–Miyazawa three-nucleon force (3NF)^70^ were added in the form of the effective NN interaction^40^. The Fujita–Miyazawa force represents the effects of the virtual excitation of a nucleon to a Δ baryon by pion-exchange processes and includes the effects of Δ-hole excitations, but does not include other effects, such as contact (*c*_D_ and *c*_E_) terms.

In this study, we explicitly treat neutrons only, whereas the protons remained confined to the ^16^O closed-shell core. As such, there is no proton–neutron interaction between active nucleons, and the neutron–neutron interaction is weaker. As this increases the relative importance of the effects from 3NF, we use the more modern 3NF of Hebeler et al.^43^, which is expected to have finer details and improved properties. We obtain effective NN interactions from this 3NF first by deriving density-dependent NN interactions from them^71^ and then by having the density dependence integrated out with the normal density. It was suggested that this 3NF produces results similar to those reported in ref. ^32^ for the F, Ne, Na and Mg isotopes. As a result of this change, the single-particle energies are shifted for the 1*d*_5/2_ and 2*s*_1/2_ orbitals by −0.72 MeV, for the 1*d*_3/2_ orbital by −0.42 MeV and for the *pf*-shell orbitals by 0.78 MeV.

### Coupled-cluster calculations and emulators

The starting point for the calculations is the intrinsic Hamiltonian,

1 $$H={T}_{{\rm{kin}}}-{T}_{{\rm{CoM}}}+{V}_{{\rm{NN}}}+{V}_{{\rm{NNN}}}.$$

Here *T*_kin_ is the kinetic energy, *T*_CoM_ the kinetic energy of the centre of mass and *V*_NN_ and *V*_NNN_ are nucleon–nucleon and three-nucleon potentials from *χ*EFT^62,72,73^ and include Delta isobars^74^. The momentum space cutoff of this interaction is Λ = 394 MeV/*c*.

We used the coupled-cluster method^75–81^ with singles-doubles and perturbative triples excitations, known as the CCSDT-3 approximation^82,83^, to compute the ground-state energy of ^28^O, and the particle-removed equation-of-motion (EOM) coupled-cluster method from refs. ^84,85^ for the ground-state energy of ^27^O. The coupled-cluster calculations start from a spherical Hartree–Fock reference of ^28^O in a model space of 13 major harmonic oscillator shells with an oscillator frequency of ħ*ω* = 16 MeV. The three-nucleon force is limited to three-body energies up to *E*_3max_ = 14ħ*ω*. For energy differences, the effects of model-space truncations and coupling to the scattering continuum are small and were neglected in the history-matching analysis.

**Extended Data Fig. 5:**
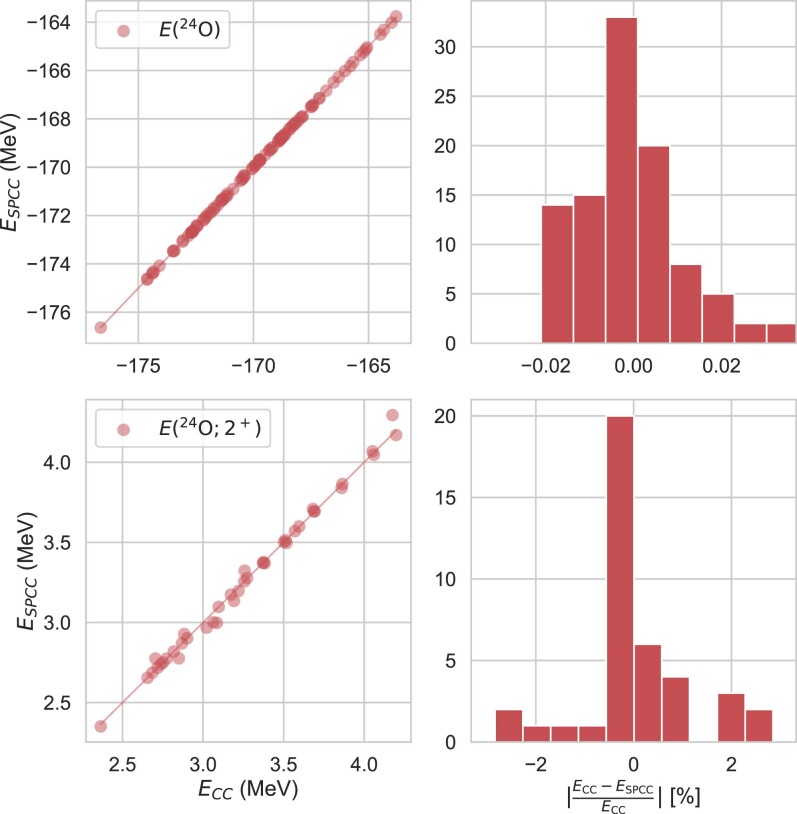
Cross-validation of emulators. Upper-left panel, total energies of ^24^O computed with the coupled-cluster method in the CCSDT-3 approximation versus the SP-CC emulator for a validation set of 100 parameter samples. Upper-right panel, distribution of residuals in percent. Lower-left panel, 2^+^ excitation energies of ^24^O computed with the coupled-cluster method in the EOM-CCSDT-3 approximation versus the SP-CC emulator for a validation set of 40 parameter samples. Lower-right panel, distribution of residuals in percent.

The LECs of this interaction are constrained by a history-matching approach using high-precision emulators enabled by eigenvector continuation^86^. These tools mimic the results of actual coupled-cluster computations but are several orders of magnitude faster to evaluate, hence facilitating comprehensive exploration of the relevant parameter space. The emulators work as follows. In the 17-dimensional space of LECs, the parameterization of the ΔNNLO_GO_(394) potential^74^ serves as a starting point around which we select emulator training points according to a space-filling lattice hypercube design for which we perform coupled-cluster computations of ground-state energies, radii and excited states of ^16,22,24^O (see Extended Data Table 1 for details). Keeping track of the variations of the observables and the corresponding coupled-cluster eigenstates as the low-energy constants are varied allows us to construct an emulator that can be used to predict the results for new parameterizations. This emulator strategy is rather general^87^ and possible because the eigenvector trajectory generated by continuous changes of the LECs only explores a relatively small subspace of the Hilbert space. Eigenvector continuation emulation tailored to coupled-cluster eigenstates is referred to as the subspace-projected coupled-cluster (SP-CC) method. In this work, we extended the SP-CC method of ref. ^88^ to excited states and increased the precision by including triples excitations by means of the CCSDT-3 and EOM-CCSDT-3 methods, respectively. Our SP-CC emulators use up to 68 training points for each observable of interest and use model spaces consisting of 11 major harmonic oscillator shells. We checked the precision of each emulator by performing emulator diagnostics^89^: confronting the emulator predictions with the results of actual coupled-cluster computations; see Extended Data Fig. 5. Once constructed, the emulators are inexpensive computational tools that can precisely predict the results for virtually arbitrary parameterizations of the EFT potentials. This allows us to explore several hundred million parameterizations with the computational cost of only a few hundred actual coupled-cluster computations. The use of emulation hence represents a critical advance, which facilitates a far deeper analysis of the coupled-cluster method that was previously infeasible owing to the substantial computational expense of the coupled-cluster calculations. Hence, these techniques overcome a substantial barrier to the use of such coupled-cluster methods.

### Coupled-cluster calculations: linking models to reality

We describe the relationship between experimental observations, *z*, and ab initio model predictions *M*(*θ*), in which *θ* denotes the parameter vector of the theoretical model, as

2 $$z=M(\theta )+{{\epsilon }}_{{\rm{method}}}+{{\epsilon }}_{{\rm{model}}}+{{\epsilon }}_{\exp }.$$

In this relation, we consider experimental uncertainties, *ϵ*_exp_, as well as method approximation errors, *ϵ*_method_. The latter represent, for example, model-space truncations and other approximations in the ab initio many-body solvers and are estimated from method-convergence studies^74^. Most notably, we acknowledge the fact that, even if we were to evaluate the model *M*(*θ*) at its best possible choice of the parameter vector, θ*, the model output, *M*(θ*), would still not be in exact quantitative agreement with reality owing to, for example, simplifications and approximations inherent to the model. We describe this difference in terms of a model discrepancy term, *ϵ*_model_. The expected EFT-convergence pattern of our model allows us to specify further probabilistic attributes of *ϵ*_model_ a priori^90–93^. We use the model errors defined in ref. ^94^. The use of emulators based on eigenvector continuation^86–88^ provides us with an efficient approximation, $\widetilde{M}(\theta )$, of the model. This approach entails an emulator error *ϵ*_emulator_ such that $M(\theta )=\widetilde{M}(\theta )+{{\epsilon }}_{{\rm{emulator}}}$, as outlined in the previous section.

Obviously, we do not know the exact values of the errors in equation (2), hence we represent them as uncertain quantities and specify reasonable forms for their statistical distributions, in alignment with the Bayesian paradigm. This allows for these uncertainties to be formally incorporated in all subsequent calculations and inferences. We also assume that the errors add independently of each other and the inputs *θ*.

### Coupled-cluster calculations: HM

In this work, we use an iterative approach for complex computer models known as HM^44–46^, in which the model, solved at different fidelities, is confronted with experimental data *z* using the relation in equation (2). The aim of HM is to estimate the set ${\mathcal{Q}}(z)$ of values for *θ*, for which the evaluation of a model *M*(*θ*) yields an acceptable—or at least not implausible (NI)—match to a set of observations *z*. HM has been used in various studies^95–97^ ranging, for example, from effects of climate modelling^98,99^ to systems biology^46^. This work represents the first application in nuclear physics. We introduce the standard implausibility measure

3 $${I}^{2}(\theta )=\mathop{\max }\limits_{i\in {\mathcal{Z}}}\frac{{\left|{\widetilde{M}}_{i}(\theta )-{z}_{i}\right|}^{2}}{{\rm{var}}\left({\widetilde{M}}_{i}(\theta )-{z}_{i}\right)},$$

which is a function over the input parameter space and quantifies the (mis-)match between our (emulated) model output ${\widetilde{M}}_{i}(\theta )$ and the observation *z*_*i*_ for all observables *i* in the target set ${\mathcal{Z}}$. This specific definition uses the maximum of the individual implausibility measures (one for each observable) as the restricting quantity. We consider a particular value for *θ* as implausible if *I*(*θ*) > *c*_*I*_ ≡ 3.0 appealing to Pukelsheim’s three-sigma rule^100^. In accordance with the assumptions leading to equation (2), the variance in the denominator of equation (3) is a sum of independent squared errors. Generalizations of these assumptions are straightforward if further information on error covariances or possible inaccuracies in our error model would become available. An important strength of the HM approach is that we can proceed iteratively, excluding regions of input space by imposing cutoffs on implausibility measures that can include further observables *z*_*i*_ and corresponding model outputs *M*_*i*_, and possibly refined emulators ${\widetilde{M}}_{i}$, as the iterations proceed. The iterative HM proceeds in waves according to a straightforward strategy that can be summarized as follows:

1. At iteration *j*: evaluate a set of model runs over the current NI volume ${{\mathcal{Q}}}_{j}$ using a space-filling design of sample values for the parameter inputs *θ*. Choose a rejection strategy based on implausibility measures for a set ${{\mathcal{Z}}}_{j}$ of informative observables.
2. Construct or refine emulators for the model predictions across the current non-implausible volume ${{\mathcal{Q}}}_{j}$.
3. The implausibility measures are then calculated over ${{\mathcal{Q}}}_{j}$, using the emulators, and implausibility cutoffs are imposed. This defines a new, smaller NI volume ${{\mathcal{Q}}}_{j+1}$ that should satisfy ${{\mathcal{Q}}}_{j+1}\subset {{\mathcal{Q}}}_{j}$.
4. Unless (i) the emulator uncertainties for all observables of interest are sufficiently small in comparison with the other sources of uncertainty, (ii) computational resources are exhausted or (iii) all considered points in the parameter space are deemed implausible, we include any further informative observables in the considered set ${{\mathcal{Z}}}_{j+1}$ and return to step 1.
5. If 4(i) or (ii) is true, we generate a large number of acceptable runs from the final NI volume ${{\mathcal{Q}}}_{{\rm{final}}}$, sampled according to scientific need.

**Extended Data Fig. 6:**
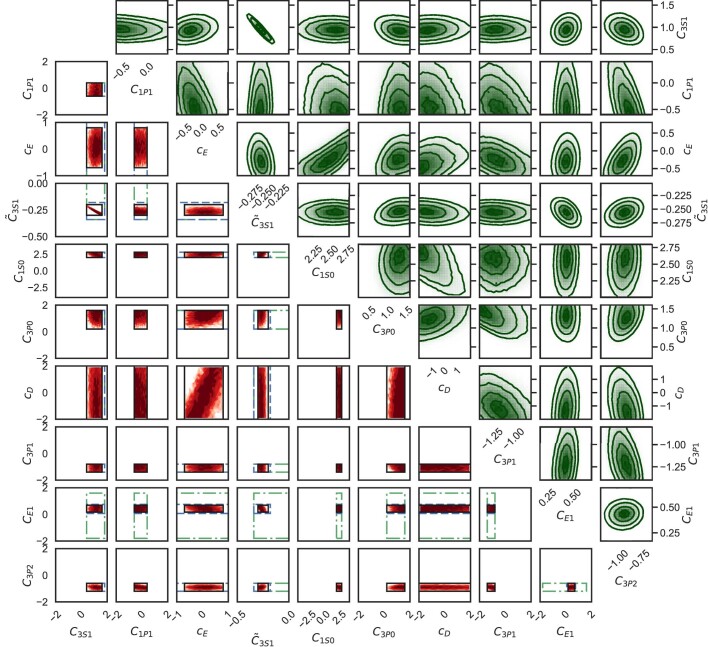
History-matching waves and Bayesian posterior sampling. Lower-left triangle, the panel limits correspond to the input volume of wave 1. The domain is iteratively reduced and the input volumes of waves 2, 3 and 4 are indicated by the green/dash-dotted, blue/dashed and black/solid rectangles, respectively. The optical depths of non-implausible samples in the final wave are shown in red, with darker regions corresponding to a denser distribution of non-implausible samples. Upper-right triangle, parameter posterior pdf from MCMC sampling with the non-implausible samples of the history-matching analysis as starting points. We use an uncorrelated, multivariate normal likelihood function and a uniform prior bounded by the first wave initial volume. Note that the relevant posterior regions are small in some directions but larger in others, such as *c*_D_ and *c*_E_.

The ab initio model for the observables we consider comprises at most 17 parameters; four subleading pion–nucleon couplings, 11 nucleon–nucleon contact couplings and two short-ranged three-nucleon couplings. To identify a set of NI parameter samples, we performed iterative HM in four waves using observables and implausibility measures as summarized in Extended Data Table 1. For each wave, we use a sufficiently dense Latin hypercube set of several million candidate parameter samples. For the model evaluations, we used fast computations of neutron–proton (*np*) scattering phase shifts and efficient emulators for the few-body and many-body observables listed. See Extended Data Table 2 for the list of included observables and key information for each wave. The input volume for wave 1 included large ranges for the relevant parameters, as indicated by the panel ranges in the lower-left triangle of Extended Data Fig. 6. In all four waves, the input volume for *c*_1,2,3,4_ is a four-dimensional hypercube mapped onto the multivariate Gaussian probability density function (pdf) resulting from a Roy–Steiner analysis of *πN* scattering data^101^. In wave 1 and wave 2, we sampled all relevant parameter directions for the set of included two-nucleon observables. In wave 3, the extra ^3^H and ^4^He observables were added. As they are known to be insensitive to the four model parameters acting solely in the *P*-wave, we therefore ignored this subset of the inputs and compensated by slightly enlarging the corresponding method errors. This is a well-known emulation procedure called inactive parameter identification^44^. For the final iteration, that is, wave 4, we considered all 17 model parameters and added a set of observables for the oxygen isotopes ^16,22,24^O and emulated the model outputs for 5 × 10^8^ parameter samples. Extended Data Fig. 6 summarizes the sequential NI volume reduction, wave-by-wave, and indicates the set *Q*_4_ of 634 NI samples after the fourth and final wave. The volume reduction is guided by the implausibility measure in equation (3) and the optical depths (see equations (25) and (26) in ref. ^46^), in which the latter are illustrated in the lower-left triangle of Extended Data Fig. 6. The NI samples summarize the parameter region of interest and can directly aid insight about interdependencies between parameters induced by the match to observed data. This region is also that in which we would expect the posterior distribution to reside. We see that the iterative HM process trains a nested series of emulators that become more and more accurate over this posterior region, as the iterations progress.

### Coupled-cluster calculations: Bayesian posterior sampling

The NI samples in the final HM wave also serve as excellent starting points for extracting the posterior pdf of the parameters *θ*, that is, *p*(*θ*|*A* = 2–24). To this end, we assume a normally distributed likelihood, according to equation (2), and a uniform prior corresponding to the initial volume of wave 1. Note that the prior for *c*_1,2,3,4_ is the multivariate Gaussian resulting from a Roy–Steiner analysis of *πN* scattering data^101^. We sample the posterior using the affine invariant Markov chain Monte Carlo (MCMC) ensemble sampler emcee^102^ and the resulting distribution is shown in the upper-right triangle of Extended Data Fig. 6. The sampling was performed with four independent ensemble chains, each with 150 walkers, and satisfactory convergence was reached (diagnosed using the Gelman–Rubin test with $| \hat{R}-1|  < 1{0}^{-4}$ in all dimensions). We performed 5 × 10^5^ iterations per walker—after an initial warmup of 5,000 steps—and kept one final sample for every 500 steps. Combining all chains, we therefore end up with 4 × 150 × 1,000 = 6 × 10^5^ final samples. Also, we explored the sensitivity of our results to modifications of the likelihood definition. Specifically, we used a Student’s *t*-distribution (*ν* = 5) to see the effects of allowing heavier tails, and we introduced an error covariance matrix to study the effect of correlations (*ρ* ≈ 0.6) between selected observables. In the end, the differences in the extracted credibility regions were not great and we therefore present only results obtained with the uncorrelated, multivariate normal distribution (see Extended Data Table 3).

**Extended Data Fig. 7:**
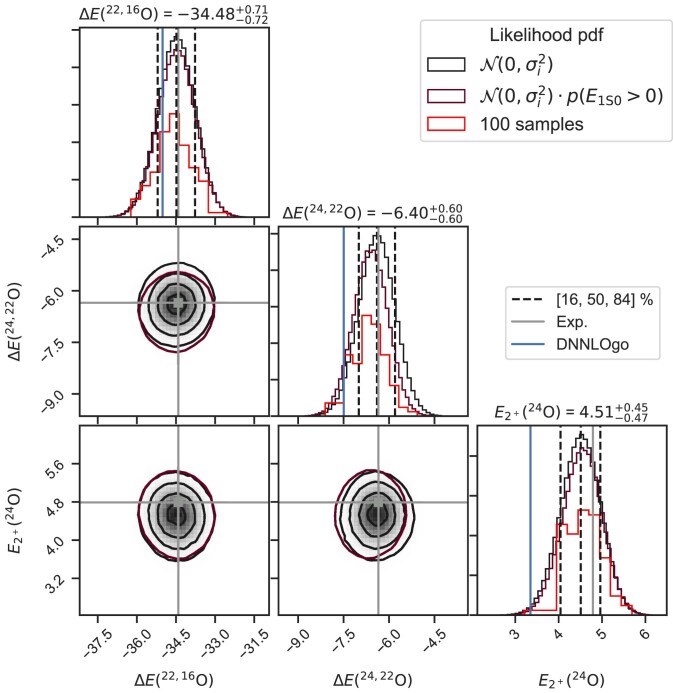
ppds for ^16,22,24^O. MCMC samples of the ppd for selected oxygen observables. The black (maroon) histogram shows results obtained with an uncorrelated, Gaussian likelihood (including a discrete probability *p*(*E*_*np*.1*S*0_ > 0|*θ*) = 1). The red histogram illustrates a low-statistics sample. The 68% credible regions and the medians are indicated by dashed lines on the diagonal, whereas the solid, vertical grey (blue) lines show the experimental target (prediction with the ΔNNLO_GO_(394) interaction).

A subset of marginal ppds is shown in Extended Data Fig. 7. Clearly, a subset of 100 samples provides an accurate low-statistics representation of this marginalized ppd. We exploit this feature in our final predictions for ^27,28^O presented in the main text. Note that the ppd does not include draws from the model discrepancy pdf. To include information about the ^25^O separation energy with respect to ^24^O, we perform a straightforward Bayesian update of the posterior pdf *p*(*θ*|*A* = 2–24) for the LECs. This complements the statistical analysis of the ab initio model with important information content from an odd and neutron-rich oxygen isotope. Using the pdf *p*(*θ*|*A* = 2–24), we draw 500 model predictions for Δ*E*(^25,24^O) and account for all independent and normally distributed uncertainties according to Extended Data Table 1. Next, we draw 121 different LEC parameterizations from the revised posterior and use coupled cluster to compute the corresponding ground-state energies of ^27,28^O. The full bivariate ppd for the ^28^O–^24^O and ^27^O–^28^O energy differences, Δ*E*(^28,24^O) and Δ*E*(^27,28^O), with associated credible regions, are shown in Extended Data Fig. 3. The effect of the continuum on the energy difference was estimated to be about 0.5 MeV in ref. ^36^ and was neglected in this work. We note that our ability to examine the full ppd for these expensive ab initio calculations provides welcome further insight, which is a direct consequence of the use of the HM procedure. We note that a sufficiently precise determination of Δ*E*(^28,24^O) and Δ*E*(^27,28^O) requires wave 4 in the HM and also using the separation energy Δ*E*(^24,25^O) for the construction of the pdf. Without input about ^25^O, the separation energy Δ*E*(^27,28^O) becomes too uncertain to be useful. It is in this sense that a sufficiently precise prediction of Δ*E*(^27,28^O) is finely tuned and cannot be based only on the properties of light nuclei up to ^4^He. Changes in the LECs that have small impact in few-nucleon systems are magnified in ^28^O. Apparently, one needs information about all nuclear shells, including the *sd* shell, to meaningfully predict this key nucleus.

### DWIA calculations

The DWIA^52,103,104^ describes proton-induced proton knockout—(*p*, 2*p*)—processes as proton–proton (*pp*) elastic scattering. This is referred to as the impulse approximation, which is considered to be valid at intermediate energies when both outgoing protons have large momenta with respect to the residual nucleus. The DWIA approach has been successful in describing proton-induced knockout reactions; in ref. ^52^, it was shown that the spectroscopic factors deduced from (*p*, 2*p*) reactions for the single-particle levels near the Fermi surface of several nuclei are consistent with those extracted from electron-induced (*e*, *e*′*p*) reactions. The transition matrix of (*p*, 2*p*) processes within DWIA theory is given by *T*_*p*2p_ = ⟨*χ*_1_*χ*_2_|*t*_*pp*_|*χ*_0_*ϕ*_*p*_⟩, in which *χ*_*i*_ are the distorted waves of the incoming proton (0) and the two outgoing protons (1 and 2), whereas *ϕ*_*p*_ is the normalized bound-state wavefunction of the proton inside the nucleus. The *pp* effective interaction is denoted by *t*_*pp*_, the absolute square of which is proportional to the *pp* elastic cross-section. The non-locality corrections^105^ to both *χ*_*i*_ and *ϕ*_*p*_ are taken into account, as well as the Møller factor^106^ for *t*_*pp*_ that guarantees the Lorentz invariance of the *pp* reaction probability. The (*p*, 2*p*) cross-section is given by *F*_kin_*C*^2^*S*|*T*_*p*2*p*_|^2^, with *F*_kin_ being a kinetic factor and *C*^2^*S* the spectroscopic factor.

In this study, the cross-section integrated over the allowed kinematics of the outgoing particles was calculated. We used the Franey–Love parameterization^107^ for *t*_*pp*_ and the Bohr–Mottelson single-particle potential^108^ to compute *ϕ*_*p*_. We have used two types of the one-body distorting potential to obtain *χ*—specifically, the Dirac phenomenology (set EDAD2 (ref. ^109^)) and a microscopic folding model potential based on the Melbourne *G*-matrix interaction^110^ and one-body nuclear densities calculated with the Bohr–Mottelson single-particle model^108^. It was found that the difference in the (*p*, 2*p*) cross-sections calculated with the two sets of distorting potentials was at most 7.5%. Also, they give almost identical shapes for the momentum distributions. As such, we have used here the average value of the cross-sections for each single-particle configuration.

## Online content

Any methods, additional references, Nature Portfolio reporting summaries, source data, extended data, supplementary information, acknowledgements, peer review information; details of author contributions and competing interests; and statements of data and code availability are available at 10.1038/s41586-023-06352-6.

### Source data

Source Data Fig. 2

Source Data Extended Data Fig. 2

Source Data Extended Data Fig. 4

## Figures and Tables

**Extended Data Table 1:**
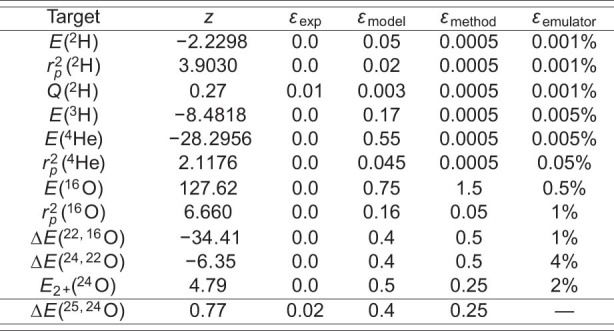
Error assignments used in the statistical analysis

Energies in MeV, squared point-proton radii in fm^2^ and the deuteron quadrupole moment in *e*^2^ fm^2^. Δ*E*(^25,24^O) is used in the Bayesian update step and the experimental target is from Hoffman et al.^24^. This observable is computed in particle-attached coupled-cluster theory^84^ and does not involve an emulator.

**Extended Data Table 2:**
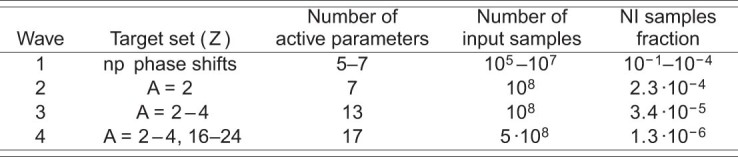
Definitions of history-matching waves used in this work

The *np* phase shifts correspond to six targets (*T*_lab_ = 1, 5, 25, 50, 100 and 200 MeV) for ^1^*S*_0_, ^3^*S*_1_, ^1^*P*_1_, ^3^*P*_0_, ^3^*P*_1_ and ^3^*P*_2_ partial waves. The *A* = 2 observables are *E*(^2^H), *r*_*p*_(^2^H) and *Q*(^2^H), whereas *A* = 3 and 4 are *E*(^3^H), *E*(^4^He) and *r*_*p*_(^4^He). Finally, *A* = 16–24 correspond to *E*(^16^O), *r*_*p*_(^16^O), the binding-energy differences Δ*E*(^24,22^O), Δ*E*( ^22,16^O) and *E*_2+_(^24^O).

**Extended Data Table 3:**
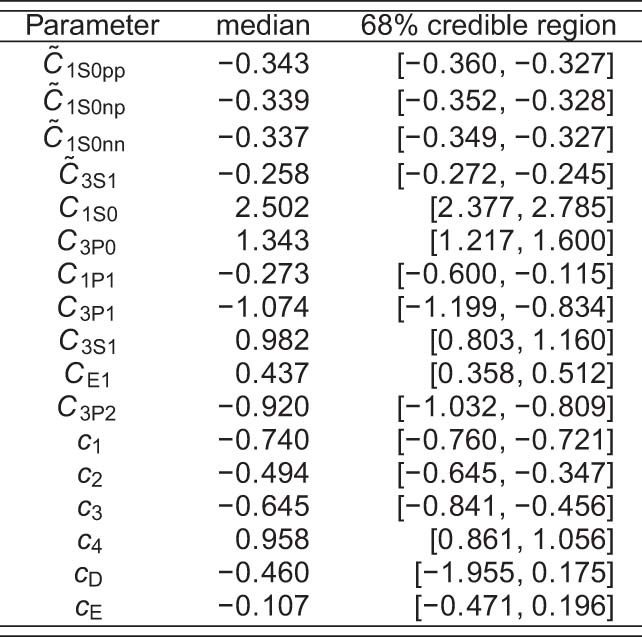
Medians and 68% credible regions (highest-density intervals) from the Bayesian posterior pdf of *χ*EFT model parameters obtained with MCMC sampling as described in the text

## Data Availability

Source data for Fig. 2a–c and Extended Data Figs. 2a–f and 4 are provided with this paper. All of the other relevant data that support the findings of this study are available from the corresponding author on reasonable request.

## Code availability

Our unpublished computer codes used to generate the results reported in this paper are available from the corresponding author on reasonable request.
