# Publisher Correction: First observation of ^28^O

**DOI:** 10.1038/s41586-023-06815-w

**Published:** 2023-11-07

**Authors:** Y. Kondo, N. L. Achouri, H. Al Falou, L. Atar, T. Aumann, H. Baba, K. Boretzky, C. Caesar, D. Calvet, H. Chae, N. Chiga, A. Corsi, F. Delaunay, A. Delbart, Q. Deshayes, Zs. Dombrádi, C. A. Douma, A. Ekström, Z. Elekes, C. Forssén, I. Gašparić, J.-M. Gheller, J. Gibelin, A. Gillibert, G. Hagen, M. N. Harakeh, A. Hirayama, C. R. Hoffman, M. Holl, A. Horvat, Á. Horváth, J. W. Hwang, T. Isobe, W. G. Jiang, J. Kahlbow, N. Kalantar-Nayestanaki, S. Kawase, S. Kim, K. Kisamori, T. Kobayashi, D. Körper, S. Koyama, I. Kuti, V. Lapoux, S. Lindberg, F. M. Marqués, S. Masuoka, J. Mayer, K. Miki, T. Murakami, M. Najafi, T. Nakamura, K. Nakano, N. Nakatsuka, T. Nilsson, A. Obertelli, K. Ogata, F. de Oliveira Santos, N. A. Orr, H. Otsu, T. Otsuka, T. Ozaki, V. Panin, T. Papenbrock, S. Paschalis, A. Revel, D. Rossi, A. T. Saito, T. Y. Saito, M. Sasano, H. Sato, Y. Satou, H. Scheit, F. Schindler, P. Schrock, M. Shikata, N. Shimizu, Y. Shimizu, H. Simon, D. Sohler, O. Sorlin, L. Stuhl, Z. H. Sun, S. Takeuchi, M. Tanaka, M. Thoennessen, H. Törnqvist, Y. Togano, T. Tomai, J. Tscheuschner, J. Tsubota, N. Tsunoda, T. Uesaka, Y. Utsuno, I. Vernon, H. Wang, Z. Yang, M. Yasuda, K. Yoneda, S. Yoshida

**Affiliations:** 1 Department of Physics, Tokyo Institute of Technology, Tokyo, Japan; 2 RIKEN Nishina Center, Saitama, Japan; 3 LPC Caen UMR6534, Université de Caen Normandie, ENSICAEN, CNRS/IN2P3, Caen, France; 4 Lebanese University, Beirut, Lebanon; 5 Lebanese-French University of Technology and Applied Sciences, Deddeh, Lebanon; 6 Institut für Kernphysik, Technische Universität Darmstadt, Darmstadt, Germany; 7 GSI Helmholtzzentrum für Schwerionenforschung, Darmstadt, Germany; 8 Helmholtz Research Academy Hesse for FAIR, Darmstadt, Germany; 9 Irfu, CEA, Université Paris-Saclay, Gif-sur-Yvette, France; 10 Institute for Basic Science, Daejeon, Republic of Korea; 11 Atomki, Debrecen, Hungary; 12 ESRIG, University of Groningen, Groningen, The Netherlands; 13 Institutionen för Fysik, Chalmers Tekniska Högskola, Göteborg, Sweden; 14 Ruđer Bošković Institute, Zagreb, Croatia; 15 Physics Division, Oak Ridge National Laboratory, Oak Ridge, TN USA; 16 Department of Physics and Astronomy, University of Tennessee, Knoxville, TN USA; 17 Physics Division, Argonne National Laboratory, Argonne, IL USA; 18 Eötvös Loránd University, Budapest, Hungary; 19 Center for Exotic Nuclear Studies, Institute for Basic Science, Daejeon, Republic of Korea; 20 Department of Physics and Astronomy, Seoul National University, Seoul, Republic of Korea; 21 Department of Advanced Energy Engineering Science, Kyushu University, Fukuoka, Japan; 22 Department of Physics, Tohoku University, Miyagi, Japan; 23 Department of Physics, The University of Tokyo, Tokyo, Japan; 24 Center for Nuclear Study, The University of Tokyo, Saitama, Japan; 25 Institut für Kernphysik, Universität zu Köln, Köln, Germany; 26 Department of Physics, Kyoto University, Kyoto, Japan; 27 Department of Physics, Kyushu University, Fukuoka, Japan; 28 Research Center for Nuclear Physics, Osaka University, Osaka, Japan; 29 Department of Physics, Osaka City University, Osaka, Japan; 30 Grand Accélérateur National d’Ions Lourds (GANIL), CEA/DRF-CNRS/IN2P3, Caen, France; 31 Center for Computational Sciences, University of Tsukuba, Ibaraki, Japan; 32 Department of Physics, Osaka University, Osaka, Japan; 33 Facility for Rare Isotope Beams, Michigan State University, East Lansing, MI USA; 34 Department of Physics, Rikkyo University, Tokyo, Japan; 35 Advanced Science Research Center, Japan Atomic Energy Agency, Ibaraki, Japan; 36 Department of Mathematical Sciences, Durham University, Durham, UK; 37 Liberal and General Education Center, Institute for Promotion of Higher Academic Education, Utsunomiya University, Tochigi, Japan

**Keywords:** Experimental nuclear physics, Nuclear physics

Correction to: *Nature* 10.1038/s41586-023-06352-6 Published online 30 August 2023

This paper was originally published under a standard Springer Nature license (© The Author(s), under a exclusive licence to Springer Nature Limited). It is now available as an open-access paper under a Creative Commons Attribution 4.0 International license, © The Author(s). The error has been corrected in the HTML and PDF versions of the article.

